# Clear Cell Renal Cell Carcinoma with Neovascularization and Ureteral Extension in a 63-Year-Old Female Unravelled by Pelvicalyceal Penetration

**DOI:** 10.12669/pjms.40.6.8715

**Published:** 2024-07

**Authors:** Nauman Zafar, Nadeem Bin Nusrat, Sarmad Imtiaz Bajwa, Saira Imtiaz

**Affiliations:** 1 Nauman Zafar, MRCS. Department of Urology, Pakistan Kidney and Liver Institute and Research Center, Lahore, Pakistan; 2 Nadeem Bin Nusrat, FRCS, CCST. Department of Urology, Pakistan Kidney and Liver Institute and Research Center, Lahore, Pakistan; 3 Sarmad Imtiaz Bajwa, MS. Department of Urology, Pakistan Kidney and Liver Institute and Research Center, Lahore, Pakistan; 4 Saira Imtiaz, M.Phil. Department of Urology, Pakistan Kidney and Liver Institute and Research Center, Lahore, Pakistan

**Keywords:** RCC, Pelvicalyceal Penetration, Neovascularization, Ureteral Extension

## Abstract

Approximately 3% to 4% of all newly diagnosed cancers are kidney tumours, which can develop from either the renal parenchymal tissue or the renal pelvis. Kidney cancer is one of the 13 most prevalent kinds of malignancy worldwide. 85% of all malignant kidney neoplasms are renal cell carcinomas (RCC). We present a rare instance of an RCC that had a thrombus in the ureter and had directly extended into the renal pelvicalyceal system.

A thorough diagnostic workup was required because the patient had a number of symptoms, including flank pain, hematuria, and weight loss. Imaging tests identified a renal parenchymal-derived infiltrative tumour with remarkable pelvicalyceal penetration. Neovascularization was found within the tumour as a result of additional search. The discovery of ureteral extension, a peculiar characteristic, raised questions regarding both local and distant metastases. R.E.N.A.L nephrometry score was 11 with high complexity. Multidisciplinary management of the intricate clinical problem was employed. After removing the tumour mass, the ureteral involvement was treated with surgical resection, adjuvant therapy, and CT monitoring over the three-month high-risk follow-up period. With surgery, targeted treatment was employed to stop the cancer from growing.

**Conclusion:**

The importance of identifying unusual RCC presentations and employing a comprehensive diagnostic and treatment strategy is emphasised by this study. The complex interaction of ureteral extension, neovascularization, and pelvicalyceal penetration highlights the aggressiveness of advanced RCC. Since we are not aware of any literature documented instances including this combination, there are few studies explaining pelvicalyceal system invasion that defies commonly recognised diagnostic and treatment paradigms for renal cell carcinoma.

## INTRODUCTION

RCC is a large, diversified group of tumours arising from renal tubular epithelial cells with a range of clinical presentations and prognostic consequences. RCC accounts for nearly 90% of all kidney malignancies detected. The classical triad of hematuria, flank pain, and palpable abdominal mass is observed in less than 10% of cases, rendering RCC a diagnostic challenge that often presents at an advanced stage.^1^ Recent advancements in imaging techniques have allowed for the detection of RCC at earlier stages, facilitating timely intervention and improved outcomes.^2^ However, certain cases deviate from the norm, exhibiting an array of intricate features that demand unique diagnostic and therapeutic approaches.^3^

To the best of our knowledge, this case presents an unprecedented combination of features in RCC, necessitating a multidisciplinary approach to both diagnosis and treatment. The intricate interplay between pelvicalyceal penetration, neovascularization, and ureteral extension challenges conventional notions of RCC behavior. In this report, we detail the clinical presentation, diagnostic workup, and management strategy for this intriguing case, contributing to the existing body of literature on atypical presentations of RCC and guiding future approaches to similarly complex cases.

### Case presentation:

**Fig.1:**
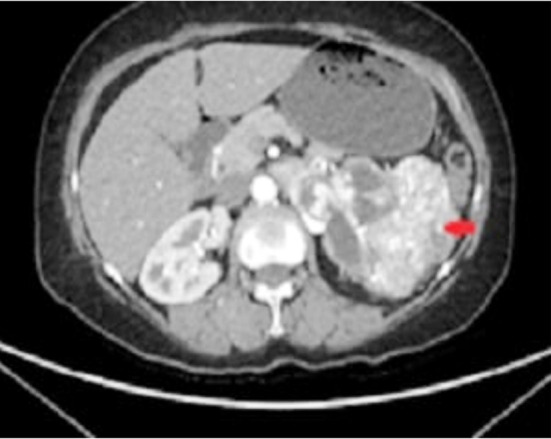
CT chest abdomen pelvis with contrast (Red arrows shows vascular space occupying lesion almost replacing the entire left kidney and invading the pelvicalyceal system, reaching the left proximal ureter.).

A 63-year-old female visited OPD first time with the active complain of left renal mass management on ultrasound and platelets 660x10^g/L. Blood pressure was119/57 mmHg, body height:149 cm, weight:57.0 kg with the BMI:25.674519 kg/m^2^. The patient was HCV positive and naswar user. Plan was made to reevaluate through CT chest abdomen pelvis with contrast with RFTS and CBC. Test values were normal while CT confirmed left RCC with invasion of pelvicalyceal system and left upper ureter with no renal venous or IVC invasion as well as no significant locoregional lymphadenopathy (Fig.1).

Her R.E.N.A.L nephrometry score was 11 with high complexity. Left lung 8 mm pulmonary parenchymal nodule abutting oblique fissure was suspicious for a metastatic deposit. Patient had ASA performance 3, underwent open left radical nephrectomy.

**Fig.2:**
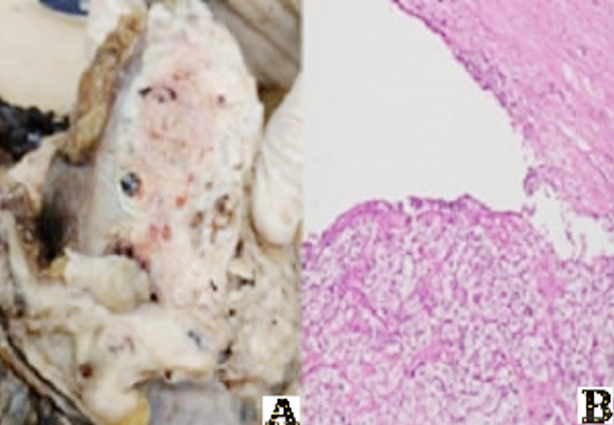
Histopathology (A) Gross with invasion of pelvis (B) Clear cell tumor in ureter. Sarcomatoid and rhambdoid features were not identified.

Under full aseptic conditions, after prep and drap, in roof top position, roof top incision made. Skin, subcutaneous tissue cut, muscles splitted layer by layer. Peritoneal identified and reflected. Gerotas opened. Left kidney approached and mobilised. The pedicle reached hemostatsis secured with endo GI stapler. Ureter ligated and cut. Speciemen retrieved that was sent for histopathology. The hemostasis was secured, and drain was placed. Ureteric stump resected again separately. Wound and muscles closed in layers with PDS (Polydioxanone suture). ASD (absorbable *skin staplers*) was done for skin. 16 Fr Foleys had already passed. Operative time was 65 minutes. Histopathology showed tumor focality as unifocal, site was whole kidney and size was 11 cm. Histologic type was clear cell renal cell carcinoma while grade was G2. Tumor was extended into pelvicalyceal system in Fig.2 (a-b)

Primary tumor was pT3a with Granulomatous inflammation. Regional lymph nodes were not involved as well as all margins are negative for invasive carcinoma. Leibovich score was 5 (intermediate risk). Immuno-Histochemistry showed CD34 as negative in lining of ureter CAIX as positive in tumor, CD10 as positive in tumor, PAX8 as positive in tumor, CK7 as negative in tumor. Previous HRCT showed moasiac pattern numerous pulmonary parenchymal nodules in the left lung, that were evaluated through Pumlonary function test. PFTs shows mild restriction (poor effort) with FEV1 1.68 and FVC 1.96 ratio 85%. It was managed through moxifloxacin for five days. No active complaints in early post op except pain at surgical site that was managed through medications. A multidisciplinary approach was employed to manage the intricate clinical scenario, to follow the high risk follow up of 3/12 months with CT and adjuvant therapy.

## DISCUSSION

The presented case of pelvicalyceal penetration of renal cell carcinoma (RCC) in a 63-years-old female patient raises several important considerations. RCC is notorious for its varied clinical presentations, and this case highlights the significance of atypical features in guiding diagnosis and management.^1^ The rarity of pelvicalyceal penetration underscores the aggressive nature of the tumor, emphasizing the importance of precise diagnostic imaging and multidisciplinary evaluation as done the same in our case. Histopathologically, clear cell RCC was confirmed, consistent with the most common subtype known for its propensity to invade surrounding structures.^4^ Renal cell carcinomas have been well treated with antiangiogenic targeted therapy drugs, which typically comprise of multikinase inhibitors. Neovascularization, which is seen inside the tumour, raises concerns regarding hematogenous spread.^5^

Notably, tyrosine kinase inhibitors, one type of targeted therapy, have demonstrated potential in reducing neovascularization and increasing survival rates in instances of advanced RCC.^6^ An all-encompassing strategy is required to address any local and distant metastases because of the exceptional characteristic in this case: ureteric extension. As seen in this instance, surgical excision continues to be a fundamental component of treatment for locally advanced RCC, enabling both final care and evaluation of histological features.^7^ Targeted medication integration after surgery can manage any remaining illness and lower the risk of recurrence.^2^ This case report adds to the need for study on complex RCC presentations and might be helpful to clinicians searching for innovative treatment strategies for uncommon cases. emphasises the importance of include the invasion of the pelvicalyceal system in the most recent, revised classification of tumour metastases, which includes RCCs with ureteral extension, neovascularization, and pelvicalyceal penetration.

## CONCLUSION

We describe a rare instance of clear cell renal cell carcinoma that directly penetrated the pelvic cavity and included granulomatous inflammation, neovascularization, and extension to the ureter. This example emphasizes the significance of identifying unusual RCC appearances and using a thorough diagnostic and therapeutic approach. The intricate interplay between pelvicalyceal penetration, neovascularization, and ureteral extension emphasizes the aggressive nature of advanced RCC. This report contributes to the existing medical literature by shedding light on an exceptional case that challenges traditional diagnostic and treatment paradigms for renal cell carcinoma.

### Authors Contribution:

**NZ:** Conceived, designed and editing of manuscript, and is responsible for integrity of research.

**NZ**, **NBN**, **SIB**, & **SI:** Did data collection and manuscript writing.

**NZ** & **SI:** Did review and final approval of manuscript.
